# Secretory Production of the *Hericium erinaceus* Laccase from *Saccharomyces cerevisiae*

**DOI:** 10.4014/jmb.2312.12043

**Published:** 2024-01-22

**Authors:** Jin Kang, Thuat Van La, Mi-Jin Kim, Jung-Hoon Bae, Bong Hyun Sung, Seonghun Kim, Jung-Hoon Sohn

**Affiliations:** 1 Synthetic Biology Research Center, Korea Research Institute of Bioscience and Biotechnology (KRIBB), Daejeon 34141, Republic of Korea; 2 Department of Biosystems and Bioengineering, KRIBB School of Biotechnology, Korea National University of Science and Technology (UST), Daejeon 34113, Republic of Korea; 3 Jeonbuk Branch Institute, Korea Research Institute of Bioscience and Biotechnology, Jeongeup 56212, Republic of Korea; 4 Cellapy Bio Inc., Daejeon 34141, Republic of Korea

**Keywords:** Recombinant protein, secretion, laccase, *Hericium erinaceus*, *Saccharomyces cerevisiae*

## Abstract

Mushroom laccases play a crucial role in lignin depolymerization, one of the most critical challenges in lignin utilization. Importantly, laccases can utilize a wide range of substrates, such as toxicants and antibiotics. This study isolated a novel laccase, named HeLac4c, from endophytic white-rot fungi *Hericium erinaceus* mushrooms. The cDNAs for this enzyme were 1569 bp in length and encoded a protein of 523 amino acids, including a 20 amino-acid signal peptide. Active extracellular production of glycosylated laccases from *Saccharomyces cerevisiae* was successfully achieved by selecting an optimal translational fusion partner. We observed that 5 and 10 mM Ca^2+^, Zn^2+^, and K^+^ increased laccase activity, whereas 5 mM Fe^2+^ and Al^3+^ inhibited laccase activity. The laccase activity was inhibited by the addition of low concentrations of sodium azide and L-cysteine. The optimal pH for the 2,2'-Azino-bis(3-ethylbenzothiazoline-6-sulfonic acid) diammonium salt was 4.4. Guaiacylglycerol-β-guaiacyl ether, a lignin model compound, was polymerized by the HeLac4c enzyme. These results indicated that HeLac4c is a novel oxidase biocatalyst for the bioconversion of lignin into value-added products for environmental biotechnological applications.

## Introduction

Plant biomass, a pivotal renewable resource for energy and biochemistry, plays a critical role in achieving carbon neutrality [1]. The conversion of plant biomass into platform molecules, facilitated by depolymerization and fermentation, is essential to make this process cost-effective, compensating for fossil fuel production and reducing greenhouse gas emissions [2]. Biorefining, a transformative process, converts plant biomass—primarily composed of cellulose, hemicellulose, and lignin-into a spectrum of marketable products and energy. While cellulose and hemicellulose are relatively easy to decompose, the robust depolymerization of lignin, owing to its rigidity and recalcitrance, necessitates advanced methods such as pyrolysis, chemical catalysis, and the involvement of natural lignin-degrading microorganisms and enzymes [3, 4].

Natural lignin-degrading microorganisms, such as fungi and bacteria, or enzymes, such as laccase (EC 1.10.3.2), lignin peroxidase (EC 1.11.1.14), manganese peroxidase (EC 1.11.1.13), and versatile peroxidase (EC 1.11.1.16), are also used to degrade lignin under mild reaction conditions [5]. Among the various lignin-degrading enzymes, laccases stand out as multicopper oxidases with the ability to oxidize a diverse range of phenolic and non-phenolic compounds [6]. Laccase is widely distributed in plants, insects, and bacteria and is involved in lignin biosynthesis by cleaving the C-C and C-O bonds of lignin. In fungi, including mushrooms, laccases play a role in sporulation, fruiting body formation, melanin synthesis, plant pathogenicity, and in vivo degradation [7]. Many fungal laccase genes have been discovered, heterologously expressed, and characterized. However, more effective enzymes must be identified and produced.

While previous efforts have explored strategies like exploring new fungal strains and optimizing growth conditions for laccase production, our research centers on a novel laccase from *Hericium erinaceus*, commonly known as lion’s mane mushroom [7]. Owing to the difficulty in culturing and manipulating the genes and genomes of basidiomycetes, even with CRISPR, the use of model organisms is strongly considered. Prokaryotic model organisms such as *Pseudomonas putida*, *Escherichia coli*, and *Streptomyces* species have been used to secrete laccase and peroxidase [8-12]. Although prokaryotic model microorganisms are good at producing enzymes, eukaryotes, especially yeasts, such as *Saccharomyces cerevisiae* and *Pichia pastoris*, are better at secretory protein production [13, 14]. In particular, endocellulase, exocellulase, and β-glucosidase are secreted by yeast for the utilization of cellulosic biomass [15, 16]. In addition, engineered yeast secretes xylose isomerase and xylanase to utilize hemicellulose [17, 18]. However, relatively little research has been conducted on laccase or peroxidase secretion in yeast.

In this study, novel laccases were identified in *H. erinaceus*, and the optimal translational fusion partners for secretion in *S. cerevisiae* were screened. Secreted laccase was purified and characterized for metal ions, inhibitors, glycosylation, optimal pH, and stability. We also confirmed the polymerization of the lignin model compounds. This laccase could be a novel biocatalytic candidate for the bioconversion of lignin into value-added products or environmental biotechnological applications.

## Materials and Methods

### Strains, Media, and Chemicals

*Escherichia coli* DH5α [F- lacZM15 hsdR17(r- m-) gyrA36] was used for general recombinant DNA techniques. Constitutive expression of laccase under the control of the GAL10 promoter was performed using *S. cerevisiae* Y2805Δ*gal80* (*Mat α pep4::HIS3 prb1 can1 his3-200 ura3-52 gal80::Tc190*). New England Biolabs provided Q5 High-Fidelity DNA Polymerase, restriction endonucleases, and endoglycosidase H (Endo H). Recombinant plasmids were constructed using the In-Fusion HD Cloning Kit (Clontech, Japan) and extracted using the QIAprep Spin Miniprep Kit (Qiagen, Netherlands). Monoclonal Anti-His Tag antibody produced in mice (Sigma-Aldrich, USA) and IRDye 800CW goat anti-mouse IgG (Li-Cor, USA) were used as primary and secondary antibodies, respectively, for western blotting. The Odyssey Fc Imaging System (Li-Cor) was used to visualize the protein bands. Standard *Trametes vesicular* laccase (TvLac) was purchased from Sigma-Aldrich. Lysogeny broth (LB) containing 100 μg/ml of ampicillin was used for culturing *E. coli*. Yeast transformants were selected on a synthetic defined medium lacking uracil (SD-Ura), and the cells were cultured in Yeast Extract Peptone Dextrose (YPD) medium for protein production. Chemicals containing 2,2'-Azino-bis(3-ethylbenzothiazoline-6-sulfonic acid) diammonium salt (ABTS) was purchased from Sigma-Aldrich. ConA-Sepharose, HiTrap Q FF, and Superose 12 10/300 GL were purchased from Cytiva (USA).

### Laccase Activity Assay

The laccase activity assay was performed as previously described with slight modifications [8]. Laccase activity was determined by oxidation of ABTS at 30°C as monitored through an absorbance increment at 420 nm in phosphate buffer. To analyze the oxidative activity of the secreted laccase, 50 μl of culture supernatant was added to 950 μl of 0.2 mM citrate phosphate buffer containing 2.0 mM ABTS.

### Purification of Laccase Enzyme from Mushroom and N-Terminus Sequence Analysis

After harvesting the fruiting body of *H. erinaceus*, the residual solid phase was mixed vigorously with 10 mM Tris-HCl buffer. The resulting soluble fractions were centrifuged at 10,000 ×*g* for 30 min, and the supernatant was centrifuged again at 25,000 ×*g* for 60 min. To purify the protein, the clarified crude protein solution was concentrated using a Labscale tangential flow filtration (TFF) system equipped with a Pellicon XL cassette of Biomax 5 kDa membrane (Merck, Germany). The crude protein solution was loaded onto a DEAE-Sepharose column (2.6 × 20 cm) with 10 mM Tris-HCl buffer (pH 8.0), 1 mM phenylmethylsulfonyl fluoride (PMSF), and a protease inhibitor cocktail. After washing with buffer solution, protein fractions were eluted with a linear gradient of 0.0-1.0 M NaCl or a step gradient of 0.25 M, 0.5 M, 1.0 M, and 2.5 M NaCl. The ABTS oxidation fractions were collected, concentrated, and loaded onto a ConA-Sepharose column (1.0 × 10 cm) containing 10 mM Tris-HCl buffer (pH 8.0), 1 mM PMSF, and a protease inhibitor cocktail. After washing, proteins were eluted with 10 mM Tris-HCl buffer containing 0.2 M α-methyl mannopyranoside. Eluted protein fractions were concentrated, loaded onto a HiTrap Q Fast Flow column (0.7 × 2.5 cm), and eluted with a linear gradient of 0.0-1.0 M NaCl. ABTS oxidation fractions were pooled, concentrated, and loaded onto a Superose 12 10/300 GL (Cytiva) column equilibrated with Phosphate-buffered saline (PBS) (pH 7.4). Purified proteins were transferred onto Immobilon Polyvinylidene fluoride (PVDF) transfer membranes. N-terminal sequences were analyzed using the Edman degradation method at the eMass Analytical Laboratory (Republic of Korea).

### Secretion of *H. erinaceus* Laccase in *S. cerevisiae*

To produce recombinant laccase proteins, yeast codon-optimized laccase genes were synthesized by Bioneer (Republic of Korea). DNA fragments were amplified via PCR using the synthesized DNA as a template and an appropriate primer set (Table 1). Each amplicon was amplified again using homologous flank forward and reverse (HF and HR) primers and then cloned into YGa-TFPn shuttle vector using in vivo recombination [19]. Transformants were selected on SD-Ura plates at 30°C and cultured in test tubes containing 3 ml of YPD broth at 30°C, 200 rpm for 48 h. Then, 1 ml culture supernatant was concentrated via acetone precipitation, mixed with sodium dodecyl sulfate-polyacrylamide gel electrophoresis (SDS-PAGE) sample buffer (Bio-Rad, USA), analyzed on 12% Tris-glycine gels under denaturing conditions, and stained with Coomassie Brilliant Blue R-250 solution.

### Purification of Recombinant Laccase

Fed-batch fermentation was performed to produce recombinant laccase, as described previously [19]. A seed culture for fermentation was prepared in a 250 ml flask containing 50 ml of SD-Ura broth at 30°C. After 24 h of growth, the seed culture was transferred to 200 ml of YPD broth to prepare the preculture. In total, 250 ml of cultured seeds were inoculated into a 5 L jar fermenter (Kobiotech, Republic of Korea) with 1.75 L of the main medium (comprising 3% yeast extract, 1.5% peptone, and 2% glucose). During fermentation, the feeding medium (comprising 5% yeast extract and 30% glucose) was added manually to maintain the glucose concentration at approximately 1%, and the pH was adjusted to 5.5 using NH_4_OH. After fermentation, the culture supernatant was harvested using centrifugation at 10,000 ×*g* for 20 min. The supernatant was collected and filtered through a 0.3 μm membrane filter (Sartorius, Germany). The filtrate was concentrated 10-fold via ultrafiltration using a hollow fiber cartridge with 30 kDa nominal molecular weight cutoff (NMWC) (Cytiva). The concentrate was dissolved in 20 mM Tris-HCl buffer, pH 7.0 (buffer A). M NaCl was added to 0.5 the sodium, which was then applied to a Hisprep Fast Flow 16/10 column (Cytiva) equilibrated with Buffer A containing 0.5 M NaCl. The enzyme was eluted with a linear gradient of imidazole (0.5 M, 0–25% saturation) in buffer B. The main fractions containing laccase were tested using ABTS assay. The fractions were concentrated using a 30-kDa NMWC ultrafiltration concentrator (Amicon Ultra-15, Millipore), dialyzed against Buffer A, and used for further experiments. The purity of the enzyme was confirmed on SDS-PAGE. Purified laccase was subjected to deglycosylation with Endo H according to the manufacturer’s instructions. SeeBlue Plus2 Pre-stained Standard Ladder and PageRuler Prestained Protein Ladder (Thermo Fisher Scientific, USA) were used to determine the molecular mass.

### Enzyme Characterization

The effects of various inhibitors and organic solvents on laccase activity were determined by adding the compounds at the indicated concentrations to the assay mixture and measuring the residual activity under standard assay conditions. The effects of pH and temperature on the enzyme activity and stability were measured using 2 mM ABTS as the substrate. The optimum pH was calculated by measuring the activity at 40°C after incubation for 10 min over the pH range 2.0–9.0 using as buffers 0.1 M phosphate (pH 2.0), 0.1 M citrate-phosphate (pH 3.0–5.0), 0.1 M phosphate (pH 6.0–7.0), and 0.1 M Tris-HCl (pH 7.0–9.0). The thermostability of the laccase was estimated after 2 h incubation of the purified enzymes at temperatures ranging from 30–70°C and pH 7.0, and the residual activity under standard assay conditions was analyzed. The effects of metal ions and inhibitors on laccase activity were determined using 2 mM ABTS as the substrate in 50 mM sodium acetate buffer (pH 5.0) in the presence of metal ions or inhibitors at appropriate concentrations. All assays were performed in triplicate.

### Depolymerization of Guaiacylglycerol-β-guaiacyl ether or Kraft lignin with HeLac4c

Guaiacylglycerol-β-guaiacyl ether (GGE), its degradation product vanillin, and kraft lignin were analyzed using high-performance liquid chromatography (HPLC, Agilent 1200 HPLC system, USA). The HPLC procedure was performed by injecting fractions using a reverse-phase Eclipse XDB-C18 column (4.6 × 150 mm, 5 μm, Agilent). Gradient separation was performed from distilled water (solvent A) to methanol (solvent B) using the following conditions: flow rate 1.0 ml/min, column temperature 25°C, time 0 min-5% B, 5 min-25% B, 10 min-40% B, 30 min-50% B, time 35 min-100% B. Using a UV detector at 280 nm, authentic GGE, and vanillin were detected at 13.522 and 10.497 min, respectively. Kraft lignin was detected as multiple peaks.

## Results

### Partial Purification of the Extracellular Laccase Proteins

**Fig. 1:**
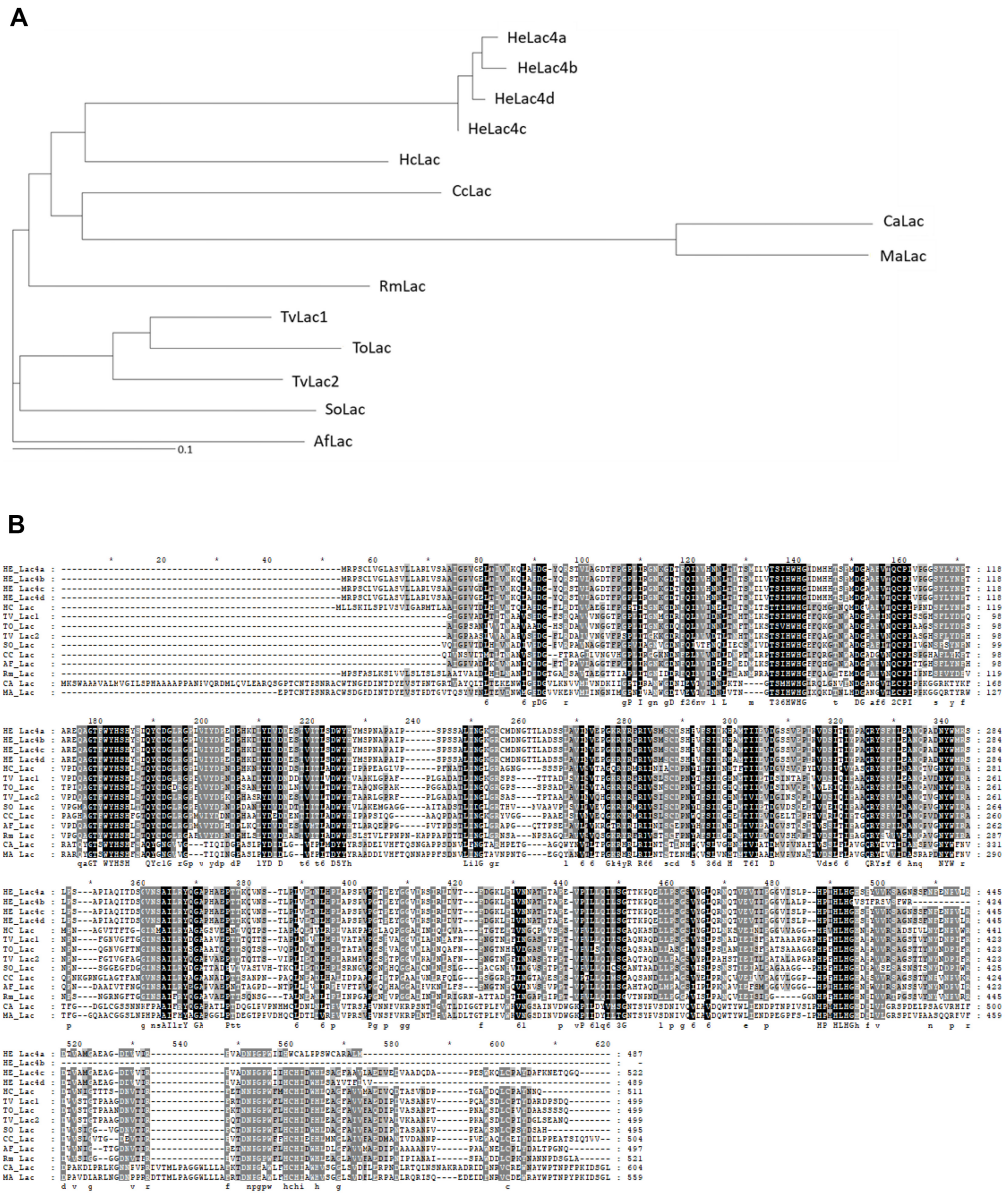
Phylogenetic analysis (**A**) and sequence alignment (**B**) of *Hericium erinaceus* laccases HeLac4a, b, c, and d with other known laccases. The conserved sequences are marked with black or gray boxes.

Mushroom *H. erinaceus* laccases were purified sequentially using a DEAE-Sepharose column, ConA-Sepharose chromatography column, HiTrap Q FF anion-exchange chromatography column, and Superose 12 size-exclusion chromatography column. Four partially purified proteins were obtained and detected as typical glycoproteins with broad diffuse bands using SDS-PAGE (data not shown). Among the four proteins, the N-terminal sequences of the 50–75 kDa protein and 37–50 kDa proteins were determined to be NH_2_-AIGPVGELTIVNKQLAPDG-CO_3_ and NH_2_-TGTISSMDDVDDAVKKCTTNINSFT VPAG-CO_3_, respectively. BLAST analysis showed the highest similarity to the laccase proteins of *Hericium alpestre* (NCBI accession number TXID135208), *Pleurotus pulmonarius* (NCBI accession number KAF4569364.1), and polygalacturonase protein of *Trametes pubescent* (NCBI accession number OJT05905.1). N-terminal sequence analysis identified the purified 50–75 kDa protein and–37–50 kDa proteins as laccase and endo-poly homologs, respectively (Fig. 1).

### Identification and Expression of Iso-Type Laccases

**Fig. 2:**
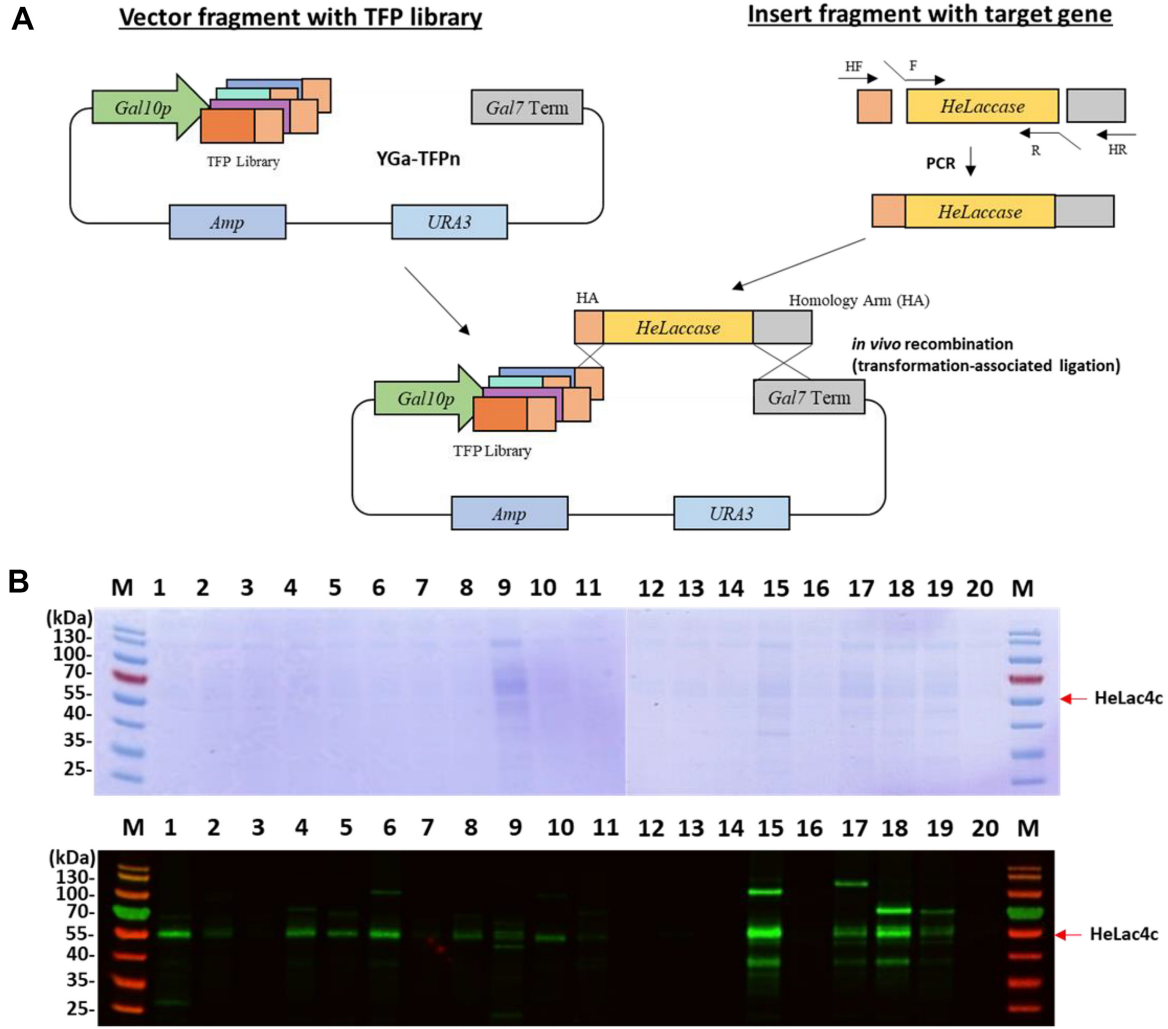
Translational fusion partner plasmid construction diagram for identification of optimum secretion fusion partner (**A**) and analysis of secretion of HeLac4c by translational fusion partners (TFPs) through SDS-PAGE (upper image) and western blot analysis (lower image) detected with anti-His antibody (**B**).

Based on the N-terminal amino acid sequence, the laccase-coding cDNAs identified in our previous RNA-seq mapping as four isotranscripts for HeLac4a, b, c, and d, among the putative laccase-coding genes in the mushroom genome were 1464, 1305, 1569, and 1470 bp in length, encoding 487, 434, 522, and 489 amino acid residues, respectively. To identify the optimal secretion fusion partner, codon-optimized laccase genes for HeLac4a, b, c, and d were combined with a previously developed translational fusion partners (TFP) library (Table 2) and secreted by *S. cerevisiae* (Fig. 2A) [14]. Among the four HeLac4 isotypes, HeLac4c alone showed oxidative activity in the presence of ABTS, guaiacol, and syringaldazine (data not shown). When fused with 20 different TFPs, HeLac4c was secreted by half, albeit at a low level (Fig. 2B). By treating Endo H after concentrating the culture supernatant of *S. cerevisiae* grown in 3 mL of YPD broth, it was challenging to visualize a clear band on SDS-PAGE (Fig. 2B, upper panel); however, secretion was confirmed using western blotting (Fig. 2B, lower panel). Among the 20 fusion partners tested, TFP15 (T15) and TFP18 (T18) were selected because of their higher expression and ABTS activity of HeLac4c (Fig. 2B). T15 is the N-terminal 120 amino acid (aa) domain of *S. cerevisiae* SPS100, which is involved in sporulation [20]. T18 is an N-terminal 134 aa segment of *S. cerevisiae* CIS3, a mannose-containing glycoprotein in the cell wall [21, 22]. The T18 were used for subsequent HeLac4c secretion experiments. Amino acid sequence analysis revealed potential copper-binding amino acids, with Cu^2+^-binding sites predicted at 417H, 474C, 479H, 484F, 84H, 86H, 420H, and 422H with SWISS-MODEL (swissmodel.expasy.org) and AlphaFold2 [23-27]. However, abnormalities in the copper-binding motifs III and IV in Lac4b and premature truncation or mutations in the final copper-binding motif IV in Lac4a and Lac4d may explain their lack of oxidative ability. HeLac4c alone retained all four Cu-binding motifs, confirming the active oxidation of ABTS. The successful expression of the novel HeLac4c laccase in yeast underscores the robustness of the yeast expression platform. Purification of HeLac4c from a 2 L Fed-batch fermentation culture supernatant yielded 2.1 mg with 465.9 U/L in total activity units.

### Characterization of HeLac4c Secreted from *S. cerevisiae*

**Fig. 3:**
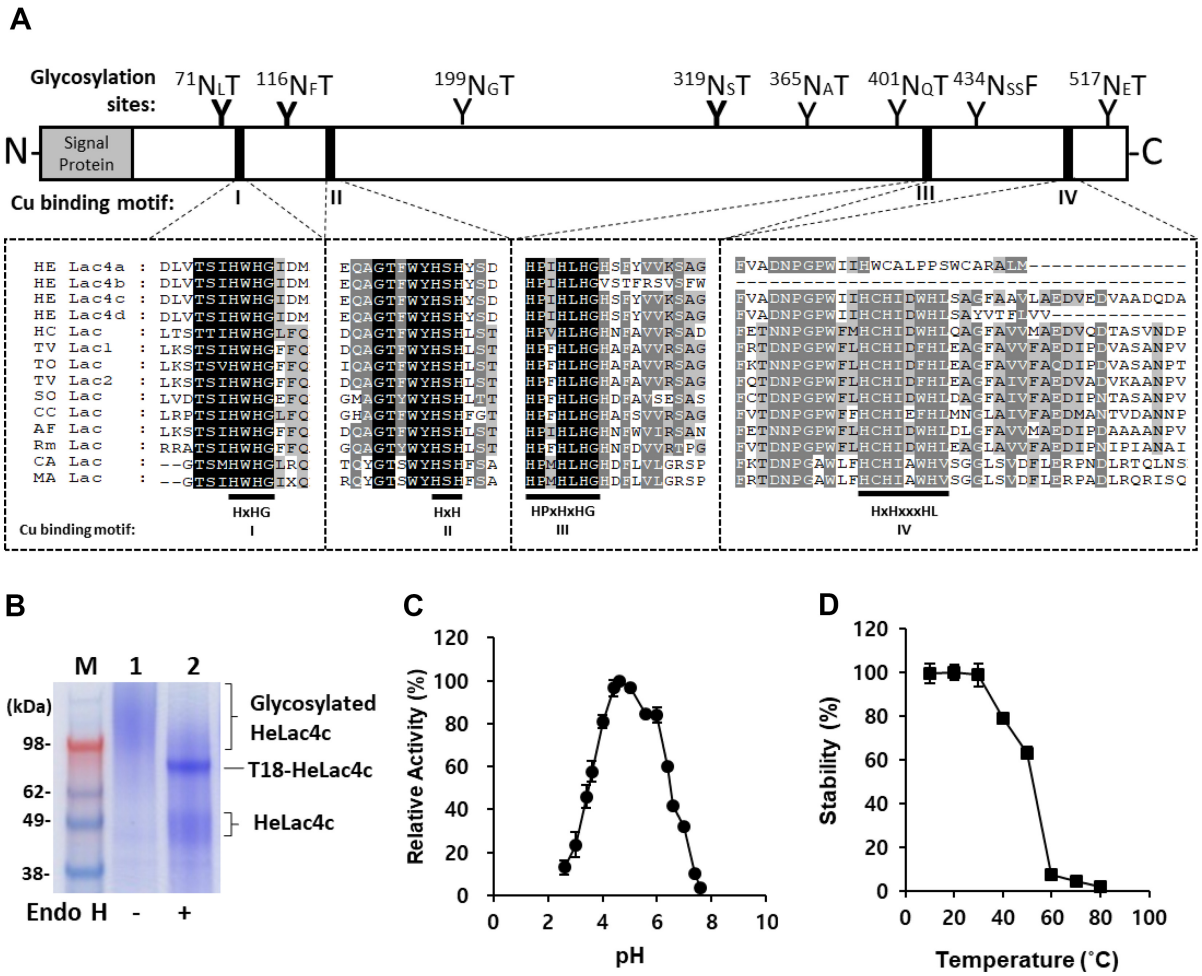
Characterization of the secreted recombinant HeLac4c enzyme. Identification of protein N-glycosylation sites and copper binding sites of laccases expressed in *S. cerevisiae* (**A**). SDS-PAGE stained with Coomassie Brilliant Blue R-250 of HeLac4c with and without Endo H treatment for the removal of N-glycosylation (**B**). Effect of pH on oxidation activities of recombinant laccase (**C**) and stability of HeLac4c after pretreatment for 2 h at 10−80°C (**D**) Activity assays of recombinant HeLac4c enzyme were performed by oxidation of ABTS in 100 mM citrate-phosphate buffer at pH 4.0.

According to the primary amino acid sequence, eight potential N-glycosylation sites (Asn-X-Ser/Thr, where X is any amino acid except proline) were identified with NetNGlyc (https://services.healthtech.dtu.dk/services/NetNGlyc-1.0/) [28] within the catalytic domain (Fig. 3A) and seven potential O-glycosylation sites were predicted with NetOGlyc. The protein secreted by T18 was purified and resolved using SDS-PAGE. In the absence of Endo H, heavily smeared bands were observed. However, upon treatment with Endo H, a clear band at approximately 70 kDa and a smeared band at approximately 55 kDa were identified (Fig. 3B). The glycoprotein nature of HeLac4c, which affects its molecular mass, was further emphasized through ABTS analysis, which showed a 25% loss of activity after removing glycosylation with Endo H, indicating that N-glycosylation of laccase could affect its binding affinity to substrates and the catalytic rate of laccase [29]. The optimum pH for ABTS oxidation was pH 4.4 (Fig. 3C), and that for syringaldazine was pH 5.0. Similar optimum pH values for ABTS and syringaldazine have been reported for other white laccases [7]. The enzyme stability after 2 h exposure at various temperatures revealed 75% retained activity until 40°C and rapid loss of activity after 50°C (Fig. 3D).

The enzyme activity of HeLac4c was measured using ABTS as the substrate at different concentrations of metal salt (0–10 mM) at pH 4.0 and 25°C (Table 3). In general, enzyme activity increased in the presence of metal salts. When 5 or 10 mM Cu^2+^ ions were added to the buffer, the ABTS oxidative activity increased by 50% compared with that in the absence of Cu^2+^. The addition of 10 mM metal ions such as Na^+^, K^+^, Mg^2+^, or Zn^2+^ increased the activity from 5 to 65%. In particular, when Na^+^ was added, the activity increased by up to 65%. For Zn^2+^ and Cr^3+^, the activity was higher when 5 mM were added than when 10 mM were added. However, metal ions such as Fe^2+^ and Al^+^ significantly inhibited HeLac4c activity. The enhancement of activity by the addition of metal ions suggests their potential use in industrial processes where metal ion exposure is expected, despite the potential limitations posed by specific metal ions such as Fe^2+^ and Al^+^.

We studied the effects of four known laccase inhibitors on HeLac4 activity (Table 4). The enzyme was incubated with different compounds prior to activity measurements. Almost complete inhibition of HeLac4c activity was observed in 1 mM sodium azide (NaN_3_), a metalloenzyme inhibitor. L-cysteine at 10 mM, which affects disulfide bond cleavage, inhibited more than 90% of the activity. Ethylenediaminetetraacetic acid (EDTA), a well-known heavy metal ion-chelating agent, inhibited the activity of HeLac4c at a concentration of 25 mM. Hydrogen peroxide (H_2_O_2_) showed the least inhibitory effect, as the enzyme retained over 50% of its original activity even at 50 mM H_2_O_2_. This high resistance to hydrogen peroxide may be attributed to structural adaptations of laccase, enabling its function as an oxidative catalyst. H_2_O_2_ is a potent oxidizing agent in the presence of Cu [30].

### Biodegradation of GGE with HeLac4c

**Fig. 4:**
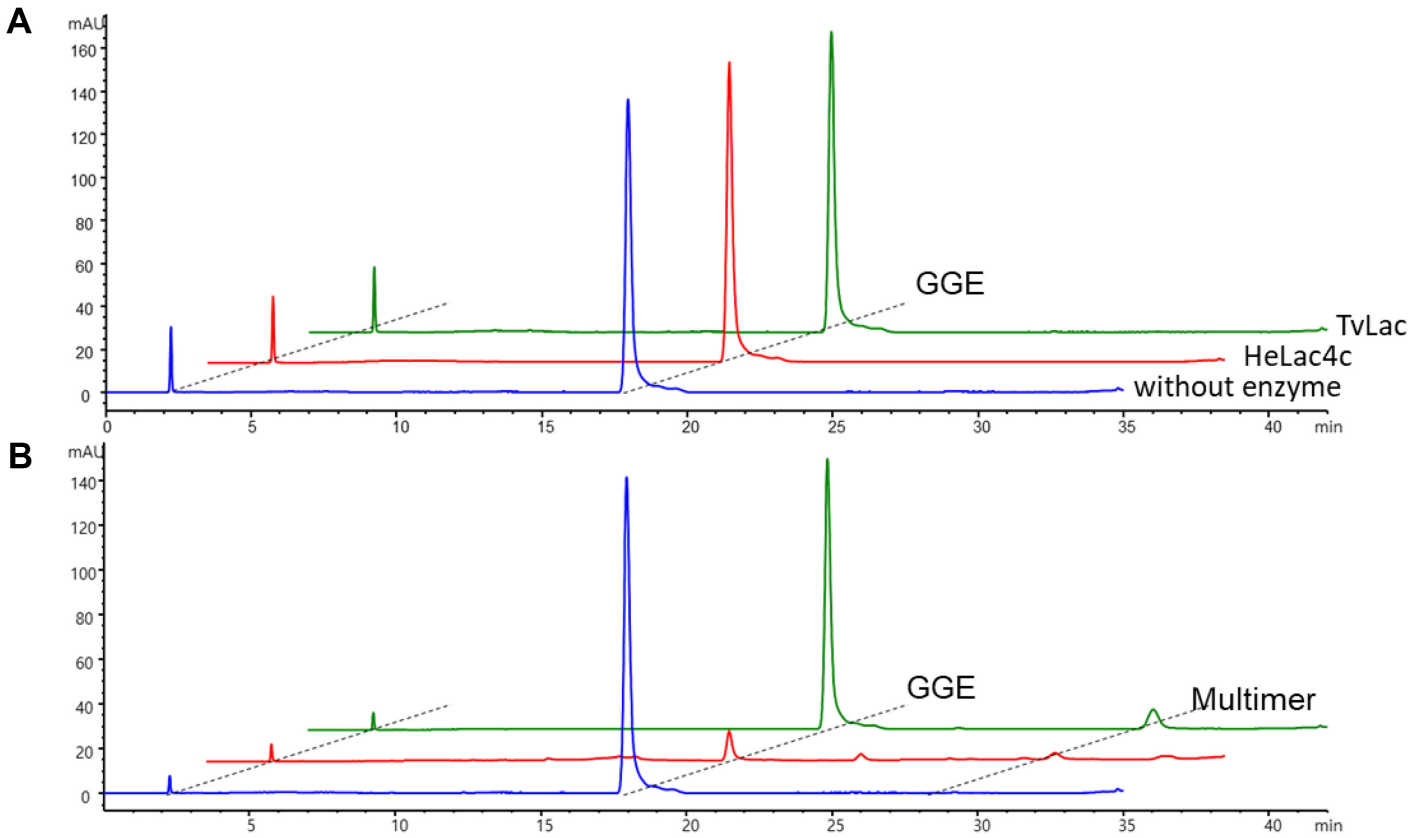
2 mM guaiacylglycerol-β-guaiacyl ether (GGE) was treated with no enzyme (blue), HeLac4c (red), and commercial TvLac (green) for testing biodegradation of laccase in pH 4 and analyzed with highperformance liquid chromatography (HPLC) without (**A**) and with addition of 1mM ABTS (**B**). GGE retention time was approximately 18 minutes, and GGE polymers at 31 and 33 minutes were observed with the addition of 1 mM ABTS. GGE depolymerized unidentified fragmented GGE monomers at 11–14 min.

Guaiacylglycerol-β-guaiacyl ether (GGE), a lignin model compound, was subjected to laccase oxidation by purified HeLac4c. Two mM GGE was treated with no enzyme (blue line in Fig. 4A), HeLac4c (red), or commercial TvLac (green) to test the biodegradation of laccase at pH 4 and analyzed using HPLC. No significant changes were observed when ABTS was not used. However, with ABTS, GGE (retention time of 18 min) was converted to GGE polymers (peaks with a retention time of ≥20 min), as previously reported [8, 31]. Trace amounts of GGE fragments (peaks with retention times between 10 and 15 min) were produced after the complete conversion of GGE to GGE polymers. Some GGE polymers formed from oxidation by HeLac4c were detected using HPLC; however, owing to the hydrophobicity of the polymers, they precipitated from the reaction solution (Fig. 4B). In addition, although HeLac4c was also incubated with Kraft lignin, no depolymerization peak was detected in HPLC analysis.

## Discussion

Four isotypes of HeLac4 were isolated from the *H. erinaceus* genome, and, similar to most fungal genomes, multiple copies of laccase-encoding genes were present. Comparison of the amino acid sequences of laccase isotypes revealed abnormalities in highly conserved laccase enzymes. HeLac4a, b, and d were inactive owing to the truncation of the C-terminal copper-binding motif, and HeLac4c alone was active. Initially, secretion of HeLac4 was attempted using mating factor alpha (MFa); however, laccase secretion was not detected. Therefore, the TFP system was used for active laccase secretion in the present study. Previous research has shown that it could improve secretion in fed-batch fermentation by 51-fold compared with the conventional TFP1(MFa) signal peptide [19]. Signal peptides are used for protein secretion; however, no single signal peptide can be used universally for all proteins [14]. To solve this problem, secretory fusion partners specific to target proteins have been identified. Protein secretion was confirmed using western blot analysis of > 50% of the 20 TFP libraries. HeLac4c was secreted when T15 or T18 were combined. It remains unclear as to why these two TFPs increased the secretion of the enzyme; they showed 2–3 times higher secretion than the other TFPs. For HeLac1a, which was previously discovered in *H. erinaceus* and expressed in *P. pastoris*, the increase in activity upon the addition of metal ions was not significant [7], whereas HeLac4c, secreted from *S. cerevisiae*, showed a metal ion-dependent increase in the activity, except for Fe and Al ions. Inhibitor assays showed that HeLac4c was more resistant to DTT than HeLac1a. HeLac1a showed minimal activity in the presence of 5 mM DTT, whereas HeLac4c retained most of its activity.

Recently, the secretion of lignin depolymerization peroxidases from *P. putida* has been reported [8]. The dye-decolorizing peroxidase is secreted using a flagellar type III secretion system. *P. putida* is a popular strain for metabolizing aromatic compounds; however, as a gram-positive bacterium, it is not very efficient at secreting proteins. However, yeasts such as *S. cerevisiae* and *P. pastoris*, which were used in this study, have excellent protein secretion capabilities. Notably, in this study, we further increased secretion efficiency from *S. cerevisiae* by searching for fusion partners specific to the target protein. Wild-type *S. cerevisiae* cannot utilize the lignin monomers produced by secreted laccases. However, integration of the pathway to convert lignin monomers to pyrocatechol and muconic acid and further integration of the beta-ketoadipate pathway can produce lignin-assimilating yeasts. Once produced, these strains will enable the consolidated bioprocessing of lignocellulosic biomass along with the previously produced cellulose- and hemicellulose-utilizing strains. This technique can be improved to a simple lignin degradation and upcycling system using a single organism containing all the lignocellulosic biomass utilization pathways.

The significance of our study lies in the discovery of a novel lignolytic enzyme and the adept ability to selectively secrete the enzyme within a model organism such as *S. cerevisiae* to supply it for utilization. Our methodology facilitates the individual secretion and comprehensive characterization of each enzyme, marking a pivotal advancement in our understanding of the enzyme. Moreover, this methodology provides a valuable platform for the production of well-characterized enzymes, creating new avenues for their application in environmental biotechnological processes and beyond. Although secretion efficiency was increased by searching for target protein-specific fusion partners, in this study, this does not guarantee mass production of proteins. One potential limitation of our study is the relatively modest size of the 20 TFP library, which, although curated from many studies. This sample size may restrict the representation of secretion signal peptides for a diverse range of proteins. Recognizing this limitation, we agree that future research endeavors should explore the expansion of the TFP library to enhance the inclusivity of signal peptides for various enzymes or amino acid lengths.

In summary, the translational fusion partner system facilitated the successful expression of novel *H. erinaceus* laccases in *S. cerevisiae*, particularly the active isotype HeLac4c. This approach allowed for efficient purification, revealing the glycoprotein nature of the recombinant laccase. The enzyme exhibits favorable characteristics, including tolerance to metal ions and resistance to inhibitors. Furthermore, HeLac4c showed promising lignolytic activity, demonstrating its potential for lignin degradation. These findings contribute to understanding laccases and suggest a novel oxidase biocatalyst for the bioconversion of lignin into value-added products for environmental biotechnological applications.

## Figures and Tables

**Table 1:** Primers used in this study to construct HeLac4 tag vectors.

Amplicon	Primer	Sequence (5’ to 3’)
HeLac4	oF1	GAATTTTTGAAAATTCAAGAATTCATGCGTCCCTCGTGCTTGG
HeLac4	F1	CTCGCCTTAGATAAAAGAGCGATTGGCCCGGTCGGCG
HeLac4a	Ra1	GTGATGGTGATGGTGATGCATCAGCGCGCGCGCACACC
HeLac4b	Rb1	GTGATGGTGATGGTGATGTCTCCAAAACGATACAGAACG
HeLac4c	Rc1	GTGATGGTGATGGTGATGCTGCCCTTGCGTCTCGTTCTTG
HeLac4d	Rd1	GTGATGGTGATGGTGATGAACCACAAGAAAGGTCACGTACG
HeLac4	HF	GGCCGCCTCGGCCTCTGCTGGCCTCGCCTTAGATAAAAGA
HeLac4	HR	GTCATTATTAAATATATATATATATATATTGTCACTCCGTTCAAGTCGAC
HeLac4His	HHR	GTCATTATTAAATATATATATATATATATTGTCACTCCGTTCAAGTCGACTTAG TGATGGTGATGGTGATG

**Table 2:** List of 20 TFPs.

TFP Number	Gene Name	Length^a^	Signal peptide Length^a^	Characteristics^b^
1	MFα	93	19	Pre-pro SS
2	YAR066	118	23	Pre-SS, N-gly, Ser, Ala-rich, GPI
3	YFR026c	130	18	Pre-SS, N-gly, TMD
4	CIS3	117	21	Pre-pro-SS, O-gly, PIR
5	SRL1	66	20	Pre-SS, N-gly, O-gly, Ser, Thr-rich
6	SIM1-1	97	19	Pre-SS, N-gly, O-gly, Ser, Ala-rich, SUN family
7	OST3	199	22	Pre-SS, O-gly
8	Yml190w	77	20	Pre-SS, N-gly, internal repeats, CWP
9	EMP24	94	19	Pre-SS, TMD
10	HSP150	174	18	Pre-pro-SS
11	ECM33	68	19	Pre-SS, GPI
12	ATG27	157	19	Pre-SS, TMD
13	UTH1	98	17	Pre-SS, SUN family, Ser-rich
14	BGL2	91	23	Pre-SS
15	SCW4	124	19	Pre-SS, CWP
16	CCW12	138	18	Pre-SS, CWP
17	FIT3	176	18	Pre-SS, GPI
18	YGP1	138	19	Pre-SS, N-gly, CWP
19	CCW14	115	22	Pre-SS, CWP
20	SED1	170	18	Pre-SS, GPI

^a^number of amino acids

^b^Pre-SS, pre-secretion signal; Pre-pro-SS, pre-pro secretion signal; N-gly, N-glycosylation site; O-gly, o-glycosylation site; GPI, glycosyl phosphatidylinositol anchor protein; PIR, protein internal repeats; CWP, cell wall protein; TMD, transmembrane domain.

**Table 3:** Effects of metal ions on the ABTS oxidation activities of HeLac4c.

Metal salt	Concentration (mM)	Relative activity (%)
None	–	100
Na_2_SO_4_	5	125
	10	165
K_2_SO_4_	5	120
	10	125
MgSO_4_	5	115
	10	120
FeSO_4_	5	5
	10	−1
ZnSO_4_	5	110
	10	105
AlK(SO_4_)_2_	5	60
	10	60
CrK(SO_4_)_2_	5	145
	10	100
CuSO_4_	5	150
	10	150

**Table 4:** Effects of inhibitors on the oxidation activities of HeLac4c.

Inhibitor	Concentration (mM)	Relative activity (%)
None	–	100
Dithiothreitol	1	96.0
	5	92.5
	10	75.9
EDTA	1	100
	5	79.9
	10	52.3
L-Cysteine	1	100
	5	26.6
	10	6.0
NaN3	0.01	100
	0.1	26.6
	1	7.0

## References

[1] Mitchard ETA (2018). The tropical forest carbon cycle and climate change. Nature.

[2] Sharma V, Tsai ML, Nargotra P, Chen CW, Sun PP, Singhania RR (2023). Journey of lignin from a roadblock to bridge for lignocellulose biorefineries: a comprehensive review. Sci. Total Environ..

[3] Coman C, Moţ AC, Gal E, Pârvu M, Silaghi-Dumitrescu R (2013). Laccase is upregulated via stress pathways in the phytopathogenic fungus *Sclerotinia sclerotiorum*. Fungal Biol..

[4] Senthivelan T, Kanagaraj J, Panda R (2016). Recent trends in fungal laccase for various industrial applications: an eco-friendly approach-a review. Biotechnol. Bioprocess Eng..

[5] Lee S, Kang M, Bae JH, Sohn JH, Sung BH (2019). Bacterial valorization of lignin: strains, enzymes, conversion pathways, biosensors, and perspectives. Front. Bioeng. Biotechnol..

[6] Pezzella C, Guarino L, Piscitelli A (2015). How to enjoy laccases. Cell. Mol. Life Sci..

[7] La TV, Sung BH, Kim S (2023). Biocatalytic characterization of *Hericium erinaceus* laccase isoenzymes for the oxidation of lignin derivative substrates. Int. J. Biol. Macromol..

[8] Lee S, Kang M, Jung CD, Bae JH, Lee JY, Park YK (2023). Development of novel recombinant peroxidase secretion system from *Pseudomonas putida* for lignin valorisation. Bioresour. Technol..

[9] Välimets S, Pedetti P, Virginia LJ, Hoang MN, Sauer M, Peterbauer C (2023). Secretory expression of recombinant small laccase genes in Gram-positive bacteria. Microb. Cell Fact..

[10] Mo Y, Lao HI, Au SW, Li IC, Hu J, Yuen HM (2022). Expression, secretion and functional characterization of three laccases in *E. coli*. Synth. Syst. Biotechnol..

[11] Wang TN, Zhao M (2017). A simple strategy for extracellular production of CotA laccase in *Escherichia coli* and decolorization of simulated textile effluent by recombinant laccase. Appl. Microbiol. Biotechnol..

[12] Xu Z, Peng B, Kitata RB, Nicora CD, Weitz KK, Pu Y (2022). Understanding of bacterial lignin extracellular degradation mechanisms by *Pseudomonas putida* KT2440 via secretomic analysis. Biotechnol. Biofuels Bioprod..

[13] De Brabander P, Uitterhaegen E, Delmulle T, De Winter K, Soetaert W (2023). Challenges and progress towards industrial recombinant protein production in yeasts: a review. Biotechnol. Adv..

[14] Bae JH, Sung BH, Kim HJ, Park SH, Lim KM, Kim MJ (2015). An efficient genome-wide fusion partner screening system for secretion of recombinant proteins in yeast. Sci. Rep..

[15] Lee CR, Sung BH, Lim KM, Kim MJ, Sohn MJ, Bae JH, Sohn JH (2017). Co-fermentation using recombinant *Saccharomyces cerevisiae* yeast strains hyper-secreting different cellulases for the production of cellulosic bioethanol. Sci. Rep..

[16] Sharma J, Kumar V, Prasad R, Gaur NA (2022). Engineering of *Saccharomyces cerevisiae* as a consolidated bioprocessing host to produce cellulosic ethanol: Recent advancements and current challenges. Biotechnol. Adv..

[17] Bae JH, Kim MJ, Sung BH, Jin YS, Sohn JH (2021). Directed evolution and secretory expression of xylose isomerase for improved utilisation of xylose in *Saccharomyces cerevisiae*. Biotechnol. Biofuels.

[18] Karaoğlan M (2023). Alternative secretory signal sequences for recombinant protein production in *Pichia pastoris*. Enzyme Microb. Technol..

[19] Ko H, Kim MJ, Kim HJ, Kang J, Lee HY, Lee JH (2023). Efficient valorization of food waste oils to renewable biodiesel by a *Candida antarctica* lipase B mutant that catalyzes the ester synthesis reaction in the presence of water. J. Clean. Prod..

[20] Percival-Smith A, Segall J (1987). Increased copy number of the 5' end of the SPS2 gene inhibits sporulation of *Saccharomyces cerevisiae*. Mol. Cell. Biol..

[21] Manning BD, Padmanabha R, Snyder M (1997). The Rho-GEF Rom2p localizes to sites of polarized cell growth and participates in cytoskeletal functions in *Saccharomyces cerevisiae*. Mol. Biol. Cell.

[22] Yin QY, de Groot PW, Dekker HL, de Jong L, Klis FM, de Koster CG (2005). Comprehensive proteomic analysis of *Saccharomyces cerevisiae* cell walls: identification of proteins covalently attached via glycosylphosphatidylinositol remnants or mild alkali-sensitive linkages. J. Biol. Chem..

[23] Bertoni M, Kiefer F, Biasini M, Bordoli L, Schwede T (2017). Modeling protein quaternary structure of homo- and hetero-oligomers beyond binary interactions by homology. Sci. Rep..

[24] Bienert S, Waterhouse A, de Beer Tjaart AP, Tauriello G, Studer G, Bordoli L (2016). The SWISS-MODEL Repository-new features and functionality. Nucleic Acids Res..

[25] Studer G, Rempfer C, Waterhouse AM, Gumienny R, Haas J, Schwede T (2019). QMEANDisCo-distance constraints applied on model quality estimation. Bioinformatics.

[26] Studer G, Tauriello G, Bienert S, Biasini M, Johner N, Schwede T (2021). ProMod3-A versatile homology modelling toolbox. PLoS Comput. Biol..

[27] Waterhouse A, Bertoni M, Bienert S, Studer G, Tauriello G, Gumienny R (2018). SWISS-MODEL: homology modelling of protein structures and complexes. Nucleic Acids Res..

[28] Gupta R, Brunak S (2002). Prediction of glycosylation across the human proteome and the correlation to protein function. Pac. Symp. Biocomput..

[29] Xu G, Wu Y, Zhang Y, Fang W, Xiao Y, Fang Z (2019). Role of *N*-glycosylation on the specific activity of a *Coprinopsis cinerea* laccase Lcc9 expressed in *Pichia pastoris*. J. Biosci. Bioeng..

[30] Lin TY, Wu CH (2005). Activation of hydrogen peroxide in copper(II)/amino acid/H_2_O_2_ systems: effects of pH and copper speciation. J. Catal..

[31] Min K, Yum T, Kim J, Woo HM, Kim Y, Sang BI (2017). Perspectives for biocatalytic lignin utilization: cleaving 4-O-5 and C(α)-C(β) bonds in dimeric lignin model compounds catalyzed by a promiscuous activity of tyrosinase. Biotechnol. Biofuels.

